# Topoisomerase II regulates yeast genes with singular chromatin architectures

**DOI:** 10.1093/nar/gkt707

**Published:** 2013-08-09

**Authors:** Christoforos Nikolaou, Ignacio Bermúdez, Chaysavanh Manichanh, José García-Martinez, Roderic Guigó, José E. Pérez-Ortín, Joaquim Roca

**Affiliations:** ^1^Molecular Biology Institute of Barcelona, CSIC, 08028 Barcelona, Spain, ^2^Department of Biology, University of Crete, 71409 Heraklion, Greece, ^3^Department of Genetics and ERI Biotecmed, University of Valencia, 46100 Burjassot, Spain, ^4^Centre for Genomic Regulation (CRG), 08003 Barcelona, Spain and ^5^Department of Biochemistry and Molecular Biology and ERI Biotecmed, University of Valencia, 46100 Burjassot, Spain

## Abstract

Eukaryotic topoisomerase II (topo II) is the essential decatenase of newly replicated chromosomes and the main relaxase of nucleosomal DNA. Apart from these general tasks, topo II participates in more specialized functions. In mammals, topo IIα interacts with specific RNA polymerases and chromatin-remodeling complexes, whereas topo IIβ regulates developmental genes in conjunction with chromatin remodeling and heterochromatin transitions. Here we show that in budding yeast, topo II regulates the expression of specific gene subsets. To uncover this, we carried out a genomic transcription run-on shortly after the thermal inactivation of topo II. We identified a modest number of genes not involved in the general stress response but strictly dependent on topo II. These genes present distinctive functional and structural traits in comparison with the genome average. Yeast topo II is a positive regulator of genes with well-defined promoter architecture that associates to chromatin remodeling complexes; it is a negative regulator of genes extremely hypo-acetylated with complex promoters and undefined nucleosome positioning, many of which are involved in polyamine transport. These findings indicate that yeast topo II operates on singular chromatin architectures to activate or repress DNA transcription and that this activity produces functional responses to ensure chromatin stability.

## INTRODUCTION

In eukaryotic cells, topoisomerases I and II (topo I and topo II) solve the topological constrains of DNA (1,2) and are prominent targets of anti-cancer drugs (3). Topo I relieves DNA torsional stress by cleaving one strand of the duplex, allowing the DNA to rotate around the uncleaved strand (4). Topo II removes DNA supercoils, knots and catenanes by transporting one segment of duplex DNA through a transient double-strand break in another (5,6). Topo II is essential to decatenate newly replicated DNA and facilitate chromosome segregation (2,7). Either topo I or topo II is required to relax the DNA supercoils generated during the progression of DNA and RNA polymerases (8,9). Accordingly, topo I is not essential in yeast cells, as the cross-inversion mechanism of topo II is more efficient than the strand-rotation mechanism of topo I in relaxing chromatinized DNA (10,11).

In addition to the aforementioned general roles, cellular topoisomerases have been postulated as regulators of gene expression. Proper chromatin assembly requires topoisomerase activity (12,13), and DNA topology largely influences the conformation, dissociation and reassociation of nucleosomes (14–16). Negative supercoiling enhances the formation of the transcription complex at gene promoters (17,18), whereas positive supercoiling precludes complex formation and transcription initiation (19,20). Thus, not surprisingly, topoisomerases have been found implicated in a large variety of transcriptional effects. In yeast cells, topo I and topo II bind preferentially to intergenic regions of highly active genes (21,22). In *Schizosaccharomyces pombe*, topo I facilitates nucleosome disassembly in gene promoters before transcription (23); in *Saccharomyces cerevisiae*, topo I and topo II act redundantly to facilitate recruitment of RNA polymerase II to nucleosome-free promoters (22). In *S. cerevisiae*, topo I affects the transcription of stress-inducible genes located in subtelomeric regions (24). A more recent study in *S. cerevisiae* concludes that topo I and topo II mutually modulate DNA supercoiling to maintain promoters in a state competent for transcriptional activation (25). In the case of higher eukaryotes, topo I (26) and topo II (27,28) have also been found to be associated to RNA polymerase complexes during transcription activation and elongation.

From the aforementioned studies, however, it is difficult to ascertain whether the transcriptional regulation of some gene subsets relies on topoisomerase-specific mechanisms. In most cases, topo I and topo II compensate each other, and it cannot be excluded that the observed transcriptional effects are secondary to the deregulation of upstream or neighboring processes. Moreover, as interfering with the topoisomerase activities can produce DNA strand breaks, transcription alterations could also be secondary to DNA damage (29). In this regard, the only clear evidence that a specific topoisomerase regulates gene expression is found in mammals, which have two topo II isoenzymes (topo IIα and topo IIβ). Topo IIα, which is essential for chromosome replication and segregation, facilitates RNA polymerase II transcription on chromatin templates (27), promotes activation of RNA polymerase I by facilitating pre-initiation complex formation (28) and interacts with chromatin remodeling complexes (30). Topo IIβ is instead dispensable for cell proliferation but essential for normal development (31–33). Topo IIβ physically interacts with developmentally controlled genes and up- or downregulates their transcription (34,35). Topo IIβ induces the activation of gene promoters regulated by nuclear hormone receptors (36), inhibits the transcription of genes regulated by the retinoic acid receptor alpha (37) and modulates the expression of genes involved in neuronal survival (38). Thus far, the molecular mechanisms by which topo IIβ regulates transcription are poorly understood. Likewise, it remains to be explored whether this exclusive role of topo IIβ is conserved in lower eukaryotes that have a unique topo II enzyme (i.e. yeast).

Here, we show that in *S. cerevisiae*, topo II regulates the transcriptional activation of specific gene subsets*.* To reveal this, we carried out a genomic transcription run-on (GRO) in *TOP2* and *top2-ts* strains to observe the genome-wide alterations of transcription rates after thermal inactivation of topo II. Because GRO reflects mainly RNA polymerase densities, plausible effects of topo II on transcript maturation and degradation were excluded (39,40). Moreover, as we conducted GRO immediately after topo II inactivation in yeast cells with native topo I activity, transcription alterations were not attributable to accumulation of DNA supercoiling, which requires prolonged inactivation of topoisomerase activity (11). Finally, as thermal inactivation of topo II could produce some general stress, we excluded from our analyses the genes commonly affected by environmental stress conditions (41). Following this approach, we identified a modest number of yeast genes strictly up- and downregulated after topo II inactivation. Remarkably, these genes present distinctive functional and structural trends in comparison with the genome average. These features suggest that, like topo II in mammals, topo II interacts in yeast with specific chromatin ensembles to regulate gene transcription.

## MATERIALS AND METHODS

### Yeast strains

*Saccharomyces cerevisiae* strains JCW25 (*TOP2*), JCW26 (*top2-4*), JCW27 (*Δtop1 TOP2*) and JCW28 *(Δtop1 top2-4*) are derivatives of FY251 (S288C genetic background) and have previously been described (42).

### Genome-wide transcription run-on

Yeast strains were grown at 30°C to logarithmic phase in YPD medium containing 2% glucose. Thermal inactivation of topoisomerase II was carried out by the addition of calculated volume of hot media to quickly equilibrate the cultures at 37°C. After 10-min incubation at 37°C, GRO was done as described (39). Briefly, ∼5 × 10^8^ cells were washed in cold water and permeabilized with 0.5% *N*-lauryl sarcosine sodium sulfate. Cells were incubated for 5 min at 30° C in 250 µl of transcription buffer containing 4 mM each of CTP, ATP, GTP and 12 µl of ^33^P-UTP (3000 Ci/mmol, 10 mCi/ml). *In vivo* labeled RNA was purified and hybridized (0.2−2 × 10^7 ^dpm/5 ml) on nylon filters printed using PCR-amplified whole ORF (open reading frame) sequences of *S. cerevisiae* (43). Array filters were exposed to an imaging plate (BAS-MP, FujiFilm) and were read in a phosphor-imager scanner (FLA-3000, FujiFilm). Images were quantified by using ArrayVision 7.0 software, taking the sARM density (with the corresponding subtracted background) as signal. Filters were also rehybridized with total yeast genomic DNA labeled by random priming and the signals were used to normalize GRO signals in each respective filter. All GRO experiments were performed in triplicate and their reproducibility was tested by the ArrayStat software (Imaging Research, Inc.), considering the data as independent and allowing the program to take a minimum number of two valid replicates to calculate the mean values for every gene. Median absolute deviation calculated individually for each sample set was used to filter out outlier values. Usually no >2% of the gene signals analyzed were discarded because of their inconsistency among the three replicates. Pair-wise comparisons between the independent sets of intensities yield high Pearson coefficients (*r* > 0.95), thus confirming the robustness of the GRO results. GRO data sets were stored in the GEO databases (http://www.ncbi.nlm.nih.gov/geo) with accession number GSE16673.

### Analysis of gene classes

In all, 1092 essential genes from *S. cerevisiae* were obtained from a genome-scale functional profiling (44). In addition, 1073 TATA genes were obtained from a concise data set, which was formed by taking into account the location and conservation of a TATA consensus in the gene upstream region and the gene sensitivity to TATA binding protein (45). Relative enrichments for each gene class were taken as the ratio of observed over expected genes of class X falling in the set of interest (down- or upregulated genes). Fisher’s exact test was used to assess significance of enrichment. Expression variability was calculated on the basis of the dynamic range exhibited in the normalized expression rates we obtained from 352 different data sets referring to roughly 2400 experimental conditions, as compiled by the SPELL database (46). For each up- and downregulated gene, we extracted the normalized expression rates and calculated the absolute difference between the values of the 2nd and 98th percentiles. Values at the bottom 1% and top 1% were not taken into account to minimize the effect of outliers. Transcriptional plasticity values of yeast genes were obtained from (47).

### Analysis of gene promoter size and orientation

Genome annotation with chromosomal locations and orientation of 6059 genes of *S. cerevisiae* was obtained from the Genome Browser of the University of California at Santa Cruz (http://genome.ucsc.edu). Given that the intergenic space in *S. cerevisiae* is rather confined, we assigned as gene promoter the complete intergenic region upstream of the transcription start site (TSS) for each gene. Bidirectional promoters were defined as the complete set of 812 non-overlapping divergent transcript pairs that shared a single TSS upstream region expanding for a region up to 600 bp long (∼26% of 6059 yeast genes). This limiting distance between two divergent ORF was chosen on the basis of a recent analysis of bidirectional promoters of *S. cerevisiae* (48) according to which divergent ORF pairs within <600 bp share a common nucleosome-free region (NFR) and are thus expected to be subject to the same regulatory modulations.

### Analysis of transcription factor binding

Putative transcription factor binding site locations for 126 yeast transcriptional factors (TF), supported by cross-species evidence, were obtained from (49,50). Occurrence of each regulator’s binding site in a region of −500 nt flanking the TSS of each deregulated gene was calculated in bins of 20 bp to create a 126 × 40 matrix for each case (all, down, up). Normalization was performed as *Z*-score, meaning that from each value the mean was subtracted and the remainder was divided over the standard deviation.

### Analysis of DNA sequence conservation and base pair composition

Sequence conservation was determined using phastCons scores (51) for *S. cerevisiae* as calculated on the basis of multiple genome alignments against six *Saccharomyces* species (*Saccharomyces paradoxus*, *Saccharomyces mikatae*, *Saccharomyces kudriavzevii*, *Saccharomyces bayanus*, *Saccharomyces castelli*, *Saccharomyces kluyveri*). GC content was calculated as the ratio of G + C bases over the total number of bases in sliding windows of 100 nts with a 1-nt overlap. GC content was calculated as the percentage of G + C bases over the total in windows of 100 nts with a sliding window approach at 1-nt intervals across a 1000-nt region flanking the TSS of down- and upregulated genes and compared with all yeast genes (average GC content for the yeast genome is 37.5%). Deviations from Chargaff’s second parity rule (PR2) were calculated as described in (52) using the relative differences of purine–pyrimidine normalized over their sum (A − T)/(A + T) and (G − C)/(G + C). Calculation was performed in a sliding window manner with parameters identical with the ones used in the GC-content analysis (100-nt windows sliding every 1 nt).

### Analysis of nucleosome positioning and stability

Nucleosome positions for the complete yeast genome were obtained from (53). Nucleosome spacing in genic regions was calculated as the mean distance between adjacent nucleosomes in a given region. Thus, for a gene occupied by N nucleosomes, the mean of the N-1 internucleosomal distances was taken as the average nucleosomal spacing. Average nucleosome occupancies for regions around disregulated genes were calculated as the overlap of genomic regions flanking the TSS of disregulated genes with the nucleosomal positions in bins of 10 bp. *Z*-score normalization was performed on the average gene class profiles. Unstable nucleosomes were obtained through an analysis reported by (54), which defined 4827 ‘appearing’ and 2593 ‘evicted’ nucleosomes under the condition of zero overlap between the normal and a heat-shock condition. ‘Appearing’ nucleosomes referred to those existing in the heat-shock condition but not in the normal one, whereas ‘evicted’ nucleosomes referred to those existing in the normal condition but absent after a heat shock. Fragile nucleosomes were obtained from (55), which defined 2641 nucleosomes with increased sensitivity to micrococcal nuclease. Average nucleosome occupancy profiles for ‘appearing’, ‘evicted’ and ‘fragile’ nucleosomes were calculated in a manner similar to the one used for bulk nucleosomes for a region of 500 nts flanking the TSS. Relative occupancies for ‘appearing’, ‘evicted’ and ‘fragile’ nucleosomes were presented as mean percentage of overlap of the genomic region.

### Analysis of nucleosome forming potential

Theoretical nucleosome affinity was performed with SymCurv (56), an *ab initio* algorithm based on the concept of the symmetry of DNA curvature. Briefly, SymCurv values were calculated at nucleotide resolution for a given DNA sequence as a score that corresponds to the symmetry of the predicted DNA curvature in a given window. Thus, SymCurv serves to capture a footprint of the inherent symmetrical structure of the histone octamer on nucleosomal DNA. Having pre-calculated SymCurv for the entire yeast genome, we retrieved values around the TSS for the complete set of genes as well as for all subclasses of genes being up- and downregulated by topo II inactivation. Mean profiles were presented as *Z*-score normalized values.

### Analysis of chromatin remodeling activities

Genome-wide localization of eight chromatin remodelers in *S. cerevisiae* (57) was used to assess their enrichment across deregulated genes. Full lists of genes occupied by each of the chromatin remodelers (Ioc3, Isw2, Arp5, Ino80, Rsc8, Snf2, Ioc4 and Isw1) were obtained and the relative over-representation of deregulated genes among them was assessed as enrichments over the expected values. Bootstrap *P*-values were calculated on the basis of 1000 randomized trials.

### Analysis of histone modification patterns

Enrichment of deregulated genes in histone modifications was assessed through the ChromatinDB database (58). The database has compiled published data on 22 different histone marks, which thus permits a concise interrogation of histone modification enrichment. Relative enrichment was evaluated on the basis of Wilcoxon rank sum test for both gene bodies and gene promoters.

## RESULTS

### Yeast genes deregulated on inactivation of topoisomerase II

We conducted a genomic transcription run-on (GRO) in *S. cerevisiae* to examine changes of transcription rate produced shortly after the inactivation of topo II. To do this, we used a *TOP2* control strain and its derivative *top2-ts* mutant. We cultured both strains at 30°C and, on exponential growth, we shifted them to 37°C for 10 min to inactivate the thermosensitive *top2-ts* enzyme (Supplementary Figure S1). At this moment, we permeabilized the cells, radiolabeled their nascent RNA transcripts and analyzed their signals in genomic arrays as detailed in the methods.

The alterations of transcription rates between both strains were modest but highly consistent in three biological replicates of the GRO experiment (Pearson coefficients > 0.95). Within the 10 min of topo II inactivation, 270 genes (∼5%) increased their transcription rate by >1.5-fold, and 158 genes (∼3%) decreased it <0.65-fold (Supplementary Figure S2A). These alterations were uncorrelated with the changes of RNA abundance produced after the more extended inactivation of topo II (120 min) that we reported in previous studies (11) (Supplementary Figure S2B). Prolonged inactivation of topo II stalls the progression of RNA polymerase II in a sharp transcript length dependent manner (11) and this dependence did not occur in the present study (Supplementary Figure S2C).

Preliminary examination of the gene subsets affected shortly after topo II inactivation revealed the presence of genes involved in the environmental stress response (ESR) defined by Gasch *et al.* (41). ESR includes ∼900 yeast genes (not including *TOP2*) that are commonly altered in many different stress conditions (41). To exclude these general stress genes from those more specifically regulated by topo II, we performed an overlap analysis. Although ESR genes were identified by RNA abundance, this comparison was reasonable because our previous studies had shown that transcription rates and mRNA levels are highly correlated in general stress genes (59,60). Roughly, one-third of the genes down- and upregulated by topo II inactivation were also down- and upregulated under general stress conditions (Supplementary Figure S3A). This filtering process did not change the overall GRO values observed in the original subsets of deregulated genes (Supplementary Figure S3B). Thus, we considered the subsets of non-overlapping genes (173 upregulated and 97 downregulated genes) as those strictly and readily affected by the inactivation of topo II (Supplementary Table S1).

### Functional classes of deregulated genes

**Figure 1.**
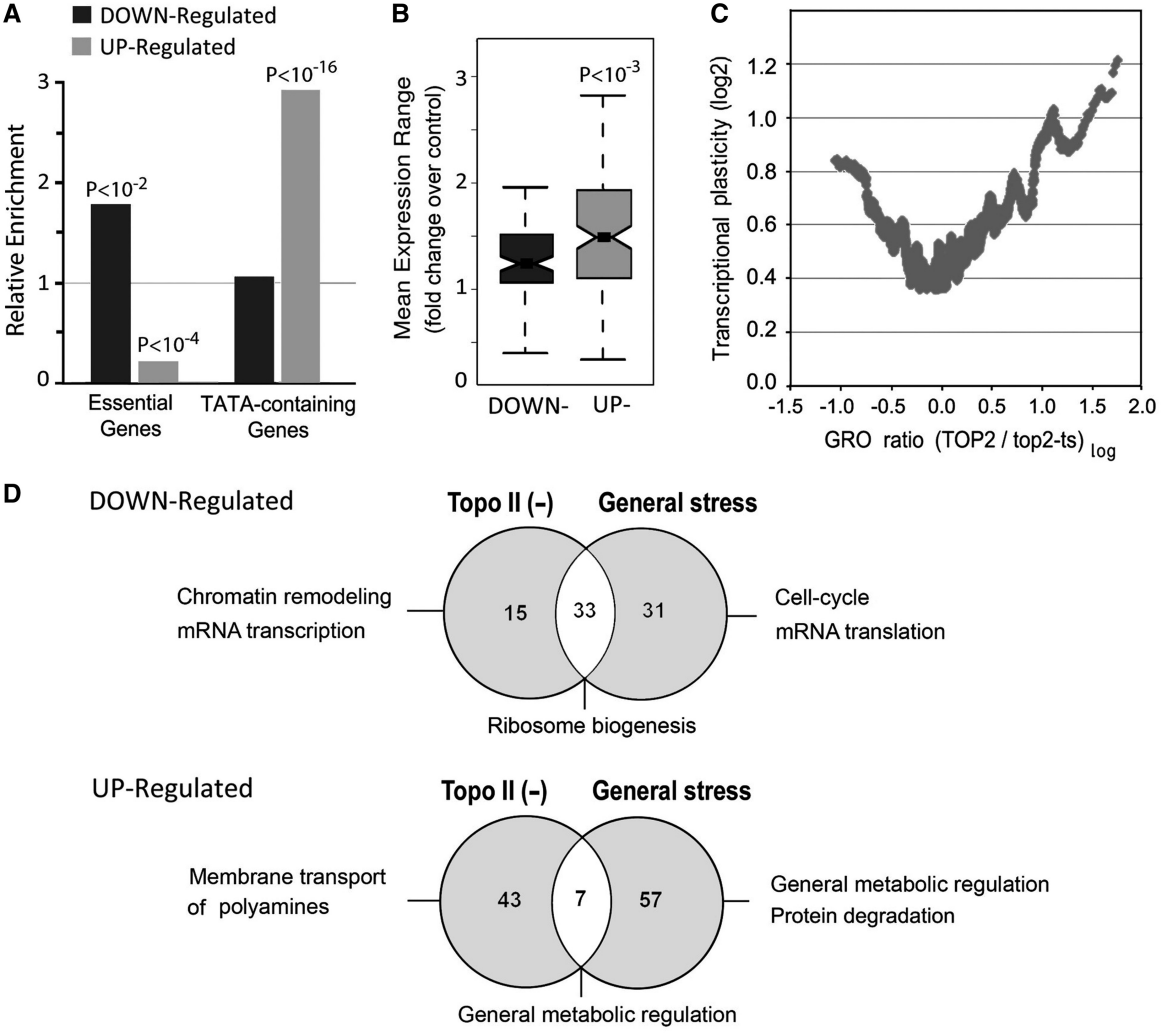
Gene classes enriched among genes deregulated by topo II inactivation. (**A**) Relative enrichment of essential, TATA-containing and chromatin-repressed genes was calculated as the ratio of observed over expected genes numbers among downregulated and upregulated genes, respectively. Bootstrap *P*-values from a 1000 randomized trials are indicated. (**B**) Mean expression variability (46) of the gene subsets deregulated on topo II inactivation. (**C**) Transcriptional plasticity of yeast genes (47) is plotted against *TOP2/top2* GRO changes (100-gene moving average). (**D**) Main functional trends of the gene subsets affected by topo II deactivation and by general stress. Trends were defined from the gene ontology categories exclusively enriched for each of the two conditions or common to both. Enriched ontology categories obtained by applying a strict *P*-value threshold of *P* < 0.001 are detailed in Supplementary Figure S2.

We went on to examine the enrichments of the gene subsets strictly deregulated by topo II among several functional categories. The upregulated subset was depleted in essential genes (<0.2-fold above the genome average) and enriched in TATA-containing genes (∼3-fold). Conversely, the downregulated subset was enriched in essential genes but not in TATA-containing genes (Figure 1A). Consistent with the above, the upregulated genes had on average a greater dynamic range of expression than the downregulated group (Figure 1B). Nonetheless, both up- and downregulated genes presented high-transcriptional plasticity relative to the genome average, thus indicating that most genes affected by topo II have a large capacity for modulated expression on changing conditions (Figure 1C).

In regard to Gene Ontology terms, as a number of functional categories could compare under general stress and under topo II inactivation, we defined ontology terms for both conditions separately, distinguishing those enriched under topo II inactivation but not under general stress. The results obtained by applying a *P*-value threshold of *P* < 0.001 in the gene enrichment are summarized in Figure 1D and the ontology categories are detailed in Supplementary Figure S4. In the case of downregulated genes, topo II inactivation and general stress were enriched in 48 and 64 Gene Ontology categories, respectively, with 33 categories common to both conditions. Downregulated functions specific for topo II deactivation were mainly related to chromatin remodeling and transcriptional regulation and contrasted thus to the downregulated functions only in general stress, which mainly affect cell division and metabolic processes. Downregulated functions common to both conditions were related to ribosome biogenesis. In the case of upregulated genes, 50 Gene Ontology categories were enriched by topo II deactivation and 64 by the general stress condition, with only 7 categories being common to both conditions. Although upregulated functions only in general stress were related to metabolism and protein degradation processes, the upregulated functions specific for topo II deactivation were mainly related to membrane transport of polyamines. Commonly upregulated functions were related to metabolic regulation.

### Promoter size and configuration for transcription factor binding at deregulated genes

**Figure 2.**
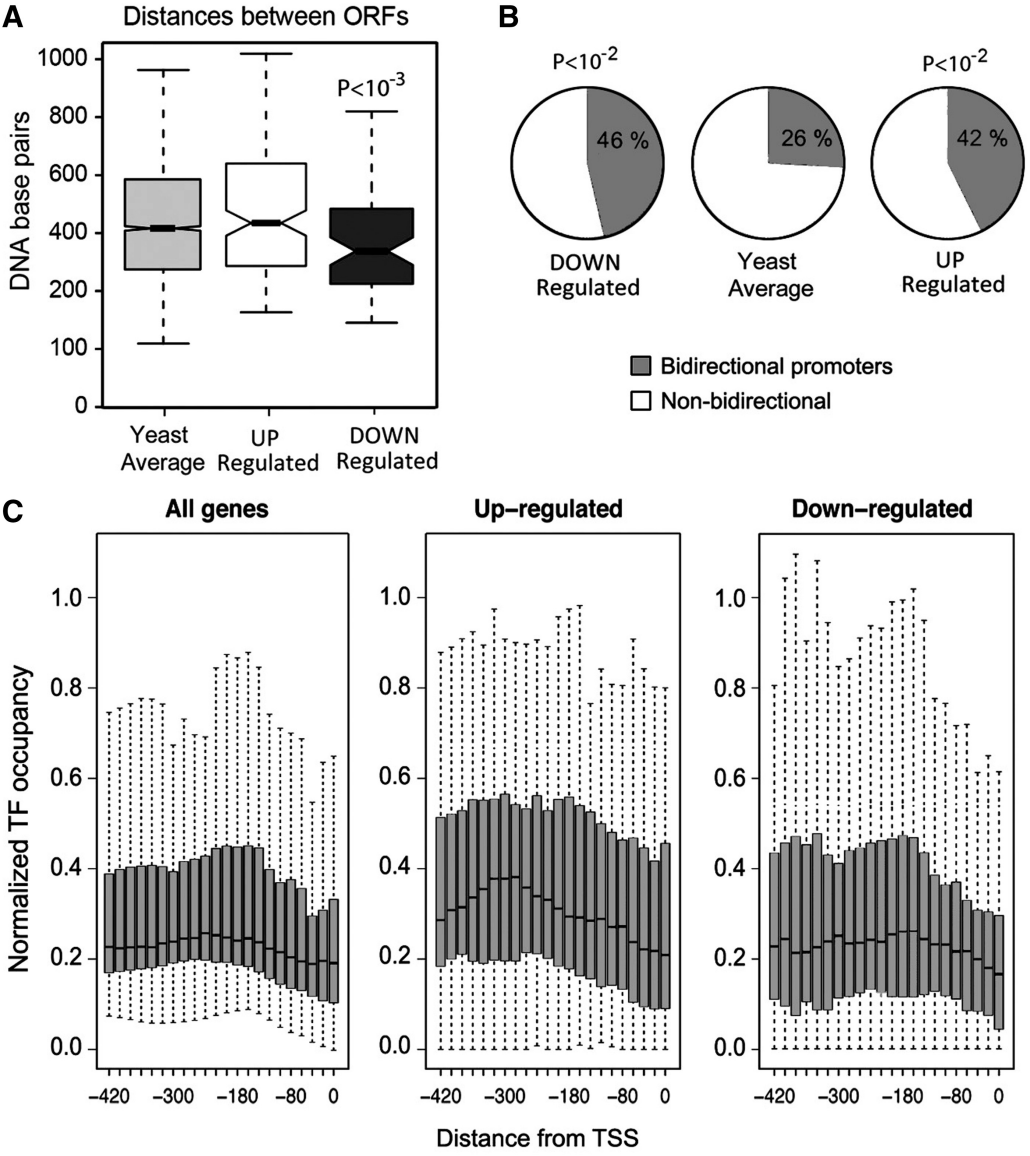
Promoter size and distribution of TFBS in genes deregulated after topo II inactivation. (**A**) Promoter size calculated as the length of the intergenic region extending upstream the TSS until the 5′ or 3′end of the most proximal gene. (B) Occurrence of bidirectional promoters defined as the intergenic regions (up to 600 bp in length) occupying the genomic space between two divergently transcribed genes (∼26% of 6059 genes). (**C**) Binding sites distribution for 126 yeast transcription factors in promoter regions of all yeast genes and the genes up- and downregulated by topo II inactivation.

The size of gene promoters in *S. cerevisiae* is constrained by the short intergenic distance between ORFs, which is <500 bp on average (40). In this regard, the subset of genes downregulated by topo II deactivation had much shorter 5′ intergenic regions (300 bp on average) than the bulk of yeast genes. Conversely, the upregulated group presented 5′ intergenic regions longer than the global average (Figure 2A). Another striking trait of these intergenic regions concerned the occurrence of divergent gene transcription, which occurs in ∼26% of the global set of yeast genes. Both up- and downregulated gene subsets were highly enriched in divergent transcription genes (42 and 46%, respectively) (Figure 2B). Moreover, some genes in the upregulated group were divergent pairs (YAL054C and YAL053W, YLL061W and YLL062C, YPR156C and YPR157W), which suggested co-regulation from their bidirectional promoter region.

We compared the distribution of binding sites for 126 putative transcription factors (TFBS) at promoter regions of up- and downregulated genes and all yeast genes (Figure 2C). Although most gene promoters presented a broad distribution of TFBS with a concentration peak around position −200, upregulated genes had much higher occupancy profile of TFBS and a concentration peak farther upstream (around position −300). Conversely, downregulated genes tended to accumulate TFBS closer to the TSS (around position −150). In regard to the occurrence of individual TFBS, we did not find significant differences between up- and downregulated genes. However, we observed complementary profiles in the binding patterns of specific transcription factors. Some TFBSs (i.e. AF2, OPI1, CAD1, PDR1, PAC, ARO80) were concentrated mainly between positions −200 and 0 in the downregulated genes, although they were distributed farther upstream in the upregulated genes (Supplementary Figure S5A). Other TFBS showed the opposite trend (i.e. GAL4, GLN3, MSN2); they were concentrated around position −200 in upregulated genes and were enriched around position −400 in downregulated genes (Supplementary Figure S5B).

### DNA sequence composition and constraint in the promoter regions of deregulated genes

**Figure 3.**
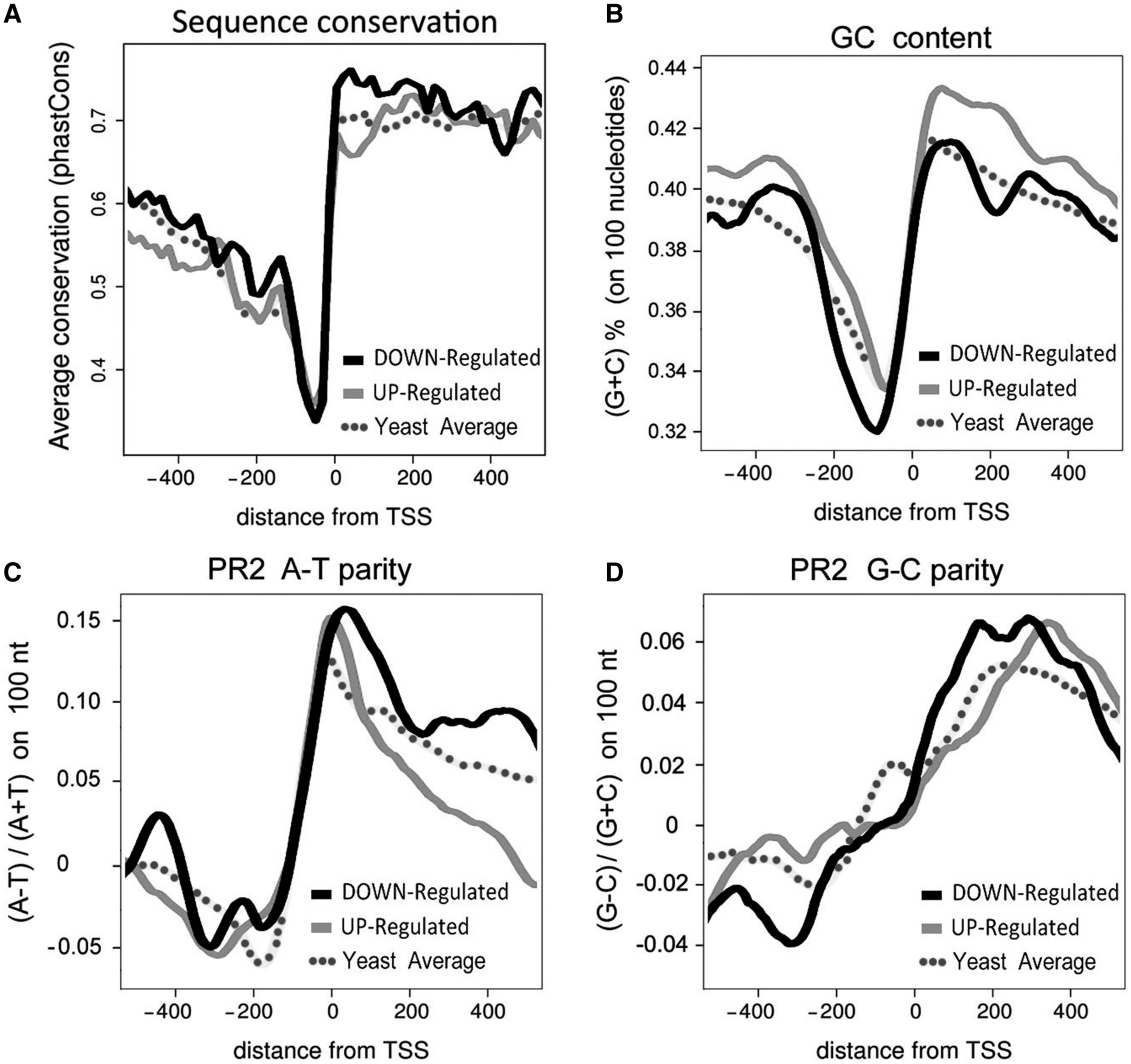
DNA base pair composition at the promoter regions of deregulated genes. (**A**) Sequence conservation calculated on the basis of multiple genome alignments against six *Saccharomyces* species. Average PhastCons score for each gene class is presented on the *y*-axis, against the relative distance from the TSS. (**B**) Average GC content calculated as the percentage of G + C bases (100 nt windows sliding every 1 nt) relative distance from the TSS (average GC content for the yeast genome is 37.5%). (**C** and **D**) PR2 violation against the distance from TSS. PR2 violations were calculated as (A − T)/(A + T) and (G − C)/(G + C) ratios (100 nt windows sliding every 1 nt).

Analysis of DNA sequence conservation, GC content and A-T/G-C parities in the promoter regions of up- and downregulated genes exposed several differences between them. First, consistent with their enrichment in essential genes, downregulated genes showed around the TSS (from position −400 to 400) a higher sequence conservation with other *Saccharomyces* species than the upregulated ones (Figure 3A). Second, although the GC content upstream of the TSS (−250 to 0) is generally reduced in all yeast genes (61), this GC-poor region was wider and deeper in the case of downregulated genes, whereas it was narrower in the upregulated ones (Figure 3B). Third, up- and downregulated genes infringed differently the PR2, according to which complementary nucleotides occur in similar frequencies (A≈T and G≈C) in each DNA strand (61). Deviations from PR2 are frequent at regulatory regions and impose prominent patterns around the sites of transcriptional initiation (62,63,51). In the case of A-T parity, deregulated subsets were similar to the genome average, presenting sharp PR2 transition from negative to positive values around the TSS and only diverging in the position of minor peaks within the region 0 to −400 (Figure 3C). In the case of G-C parity, the transition from negative to positive values around the TSS was more progressive, spanning the region −200 to +200. This transition was more sharp in the downregulated genes compared with the upregulated ones and the genome average (Figure 3D).

### Nucleosomal organization of deregulated genes

**Figure 4.**
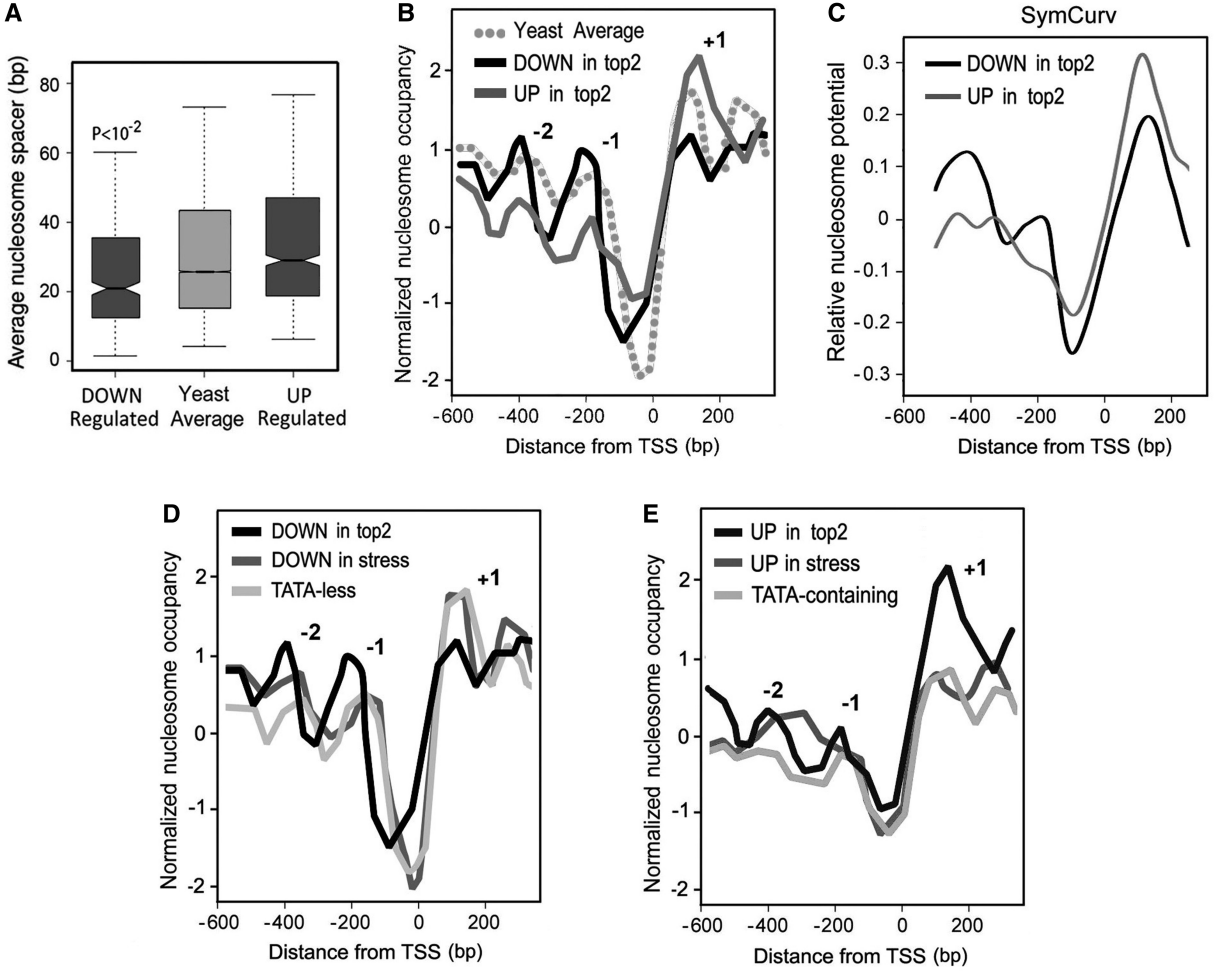
Nucleosome organization of deregulated genes. (**A**) Nucleosome spacing within each gene group was calculated as the mean distance between adjacent nucleosomes for the genic regions (see ‘Materials and Methods’ section for details). *P*-value for a *t*-test was <0.01. (**B**) Comparison of nucleosome positional landscape of yeast genes (average), and genes up- and downregulated on topo II inhibition. (**C**) Theoretical nucleosome occupancy based on SymCurv (56). Average SymCurv profiles were calculated at 1 bp resolution as described in the ‘Materials and methods’ section. Results are represented as normalized *Z*-scores. (**D**) Comparison of nucleosome positional landscape of genes downregulated on topo II inhibition, genes downregulated by general stress and TATA-less genes. (**E**) Comparison of nucleosome positional landscape of genes upregulated on topo II inhibition, genes upregulated by general stress and TATA-containing genes. Nucleosome positioning patterns were obtained based on the experimentally defined positions (53). Relative nucleosome occupancy is plotted as normalized *Z*-score values.

We examined the nucleosome landscape for up- and downregulated genes based on the experimentally defined nucleosome positions, which had been compiled in recent years from several genome-wide studies (53). One main divergence was related to the overall nucleosome density (Figure 4A). Compared with the yeast average, nucleosomes were spaced more closely in downregulated genes (average spacing 21 bp), whereas a more sparse occupancy occurred in the upregulated genes (average spacing 29 bp). However, most revealing differences regarded to nucleosome positioning nearby the TSS (Figure 4B). Both up- and downregulated subsets had NFRs upstream of the TSS, which were weaker than the average of all yeast genes. NFRs were in addition wider for downregulated genes, a fact consistent with their wider GC-depletion profile (Figure 3B). In comparison with the yeast average, positioning of nucleosomes −1 and −2 at the promoter regions was well defined for downregulated genes but it presented weak patterns in the upregulated genes. In turn, upregulated genes presented a strong positional signal for nucleosome +1 at the ORF region, although this signal was weak for the downregulated group. Remarkably, all the aforementioned divergences in nucleosome positioning strength were consistent with the nucleosome-forming potential of their DNA sequences. We assessed this potential by SymCurv, an *ab initio* method that predicts nucleosome positioning and stability through the assessment of symmetric local DNA bendability patterns (56). In downregulated genes, SymCurv predicted well-defined peaks for the positions of −1 and −2 nucleosomes, whereas in upregulated genes, the SymCurv profile revealed a more overall coarse pattern in the promoter and ORF regions with the exception of a very prominent peak for the occupancy of the +1 nucleosome (Figure 4C). To corroborate the singularity of the nucleosome positioning patterns of genes deregulated by topo II, we compared them with the positioning patterns of genes affected by general stress conditions and of TATA-containing genes. Unlike the genes downregulated after topo II inactivation, the genes downregulated by general stress and TATA-less genes did not present prominent positional peaks for nucleosomes −1 and −2 and a well-defined peak for nucleosome +1 (Figure 4D). Likewise, genes upregulated after topo II inactivation highly contrasted with genes upregulated by general stress and TATA-containing genes, which did not have a strong positional signal for nucleosome +1 (Figure 4E).

### Nucleosome dynamics and remodeling activities at the promoter region of deregulated genes

**Figure 5.**
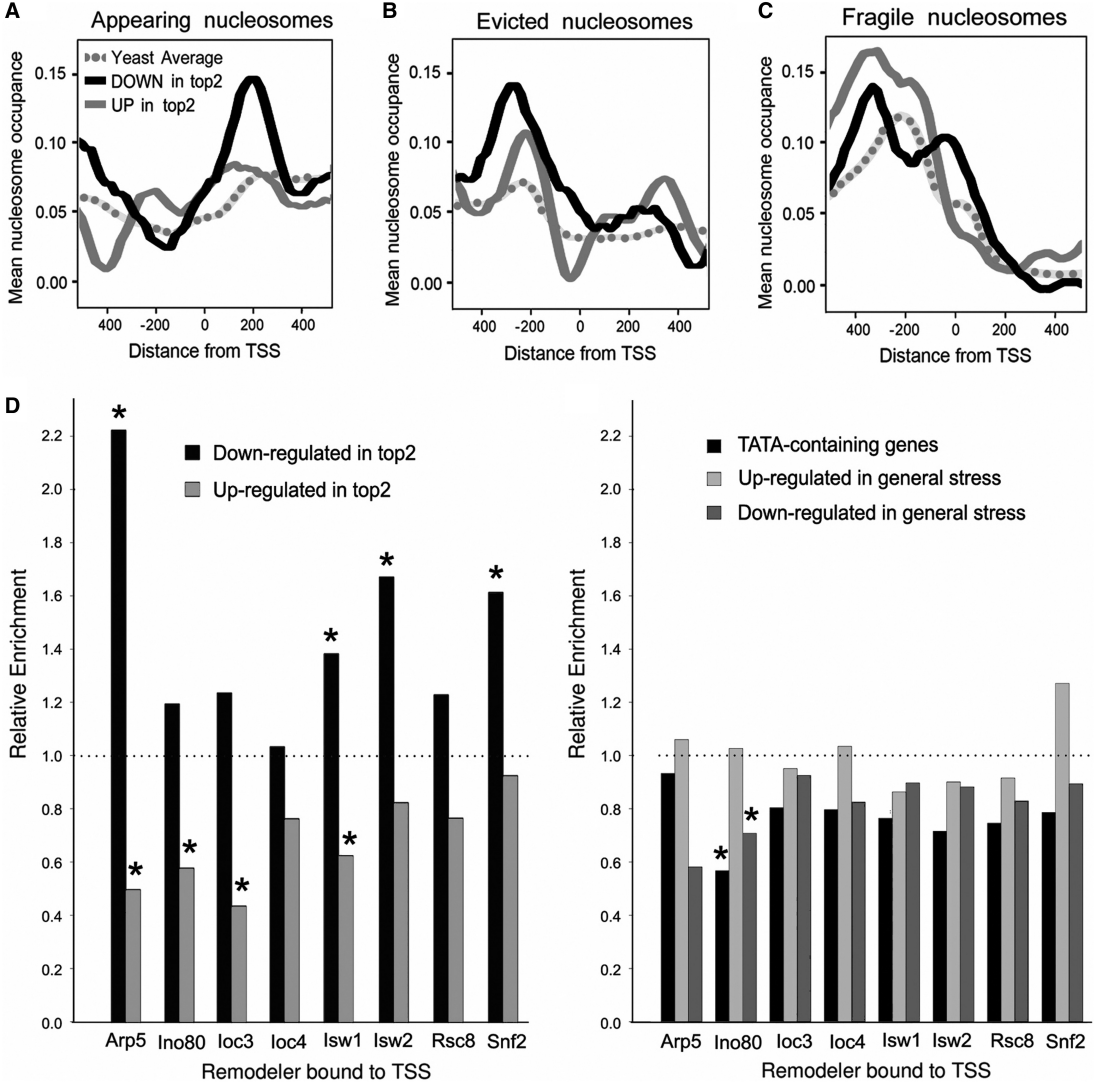
Nucleosome stability and chromatin remodelers at the promoter region of deregulated genes. (**A**) Relative occupancy of regions around the TSS of deregulated and total yeast genes by ‘appearing’ nucleosomes (54), (**B**) ‘evicted’ nucleosomes (Shivaswamy *et al.*, 2008) and (**C**) ‘fragile’ nucleosomes (55). Relative occupancies were defined as the percentage of overlap of the underlying region by a nucleosome at 10 bp resolution. (**D**) Relative enrichments/depletions of down- and upregulated genes among genes whose TSS are occupied by eight chromatin remodellers (57). Values are calculated as deviations from the expected values for the genome average (corresponding to 1). Bootstrap *P*-values were calculated on the basis of 1000 randomized gene sets. Stars denote *P* < 0.001.

In addition to nucleosome positioning, other studies have described yeast nucleosomes as ‘appearing’ or ‘evicted’ according to their presence or absence after a heat shock (54) and as ‘fragile’ based on their susceptibility to MNase digestion (55). We obtained such nucleosomes and examined their relative enrichment near the TSS in genes deregulated by topo II. Compared with the yeast average, genes deregulated by topo II presented a more dynamic nucleosomal pattern. In particular, downregulated genes showed a prominent peak for an appearing nucleosome at position +1 (Figure 5A); both up and downregulated genes presented a higher occurrence of evicted and fragile nucleosomes in positions upstream the TSS (Figure 5B and C). Recent studies have reported also the genome-wide localization of different chromatin remodelers in *S. cerevisiae* (57). Thus, we examined the association of these remodeling complexes (Arp5, Ino80, Loc3, Loc4, Isw1, Isw2, Rsc8, Snf2) with the promoter regions of the genes deregulated after topo II deactivation. The results were revealing. Relative to the yeast gene average, the downregulated genes were highly enriched in chromatin remodeler activities, whereas the contrary occurred in the upregulated group (Figure 5D). These opposite trends occurred in the eight remodelers examined and markedly contrasted with the relative enrichments observed in genes up- and downregulated during general stress and in TATA-containing genes (Figure 5E).

### Histone modification pattern of deregulated genes

**Figure 6.**
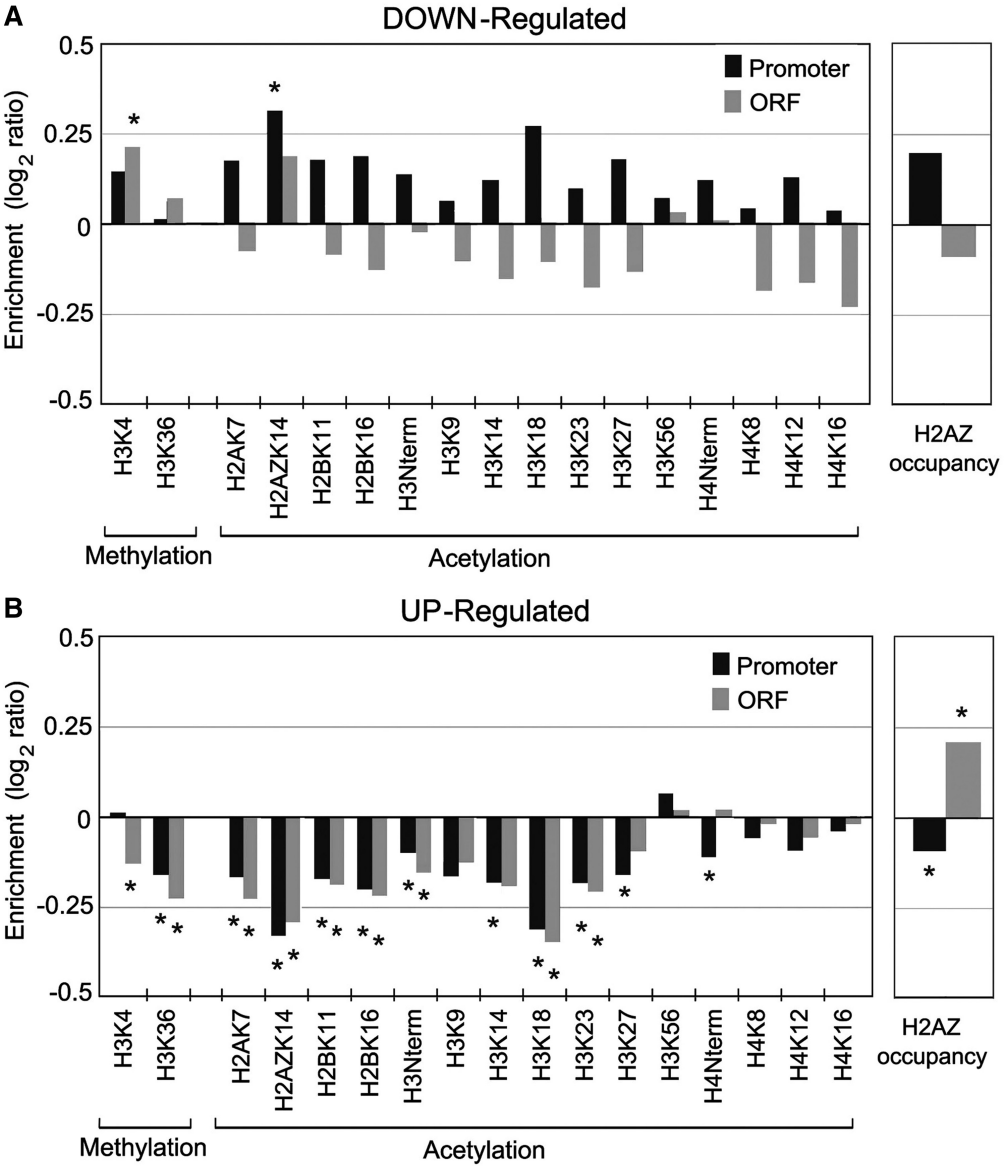
Histone modification pattern of deregulated genes. Gene subsets downregulated (**A**) and upregulated (**B**) after topo II inactivation were analyzed for histone modification enrichments in ChromatinDB (58). Relative enrichment (gene subset average compared with the total yeast average) of each indicated modification was evaluated based on a Wilcoxon rank sum test for both gene promoters and the ORF regions. Stars denote significant enrichment or depletion (*P* < 0.001).

We examined the chromatin landscapes of the genes deregulated on topo II inactivation with ChromatinDB, a web-based application that warehouses genome-wide patterns of histone modifications for *S. cerevisiae* (58). On one side, the average pattern of modifications in downregulated genes was not different from the one found in most yeast genes (Figure 6A). Only a significant increase of H2AZK14 acetylation and H3K4 methylation was observed in their promoter and ORF regions, respectively. These modifications contrasted with those typically found in genes downregulated by general stress, which tend to be hypo-acetylated at their promoter regions (Supplementary Figure S6). On the other side, upregulated genes presented a distinctive landscape compared with the genome average (Figure 6B). They exhibited a significant reduction in histone acetylation, at both the promoters and the ORF regions. Di-methylation of H3K36 was also significantly reduced in promoters and ORF regions. The occupancy of H2AZ, a histone variant commonly found in nucleosomes around the TSS, was significantly enriched downstream the TSS although depleted upstream from it. This modification pattern of upregulated genes highly contrasted also with the patterns found in genes upregulated by general stress conditions and in TATA-containing genes (Supplementary Figure S6).

## DISCUSSION

The genomic run-on conducted in this study shows that inactivation of topo II in yeast triggers quick and reproducible changes in the transcription rate of specific genes, irrespective of other processes that may affect RNA maturation and degradation. As the yeast cells retained their native topo I activity, the observed alterations are unlikely caused by the incapacity to relax DNA supercoiling during transcriptional elongation. Moreover, the fact that topo II inactivation deregulates genes of high transcriptional plasticity, downregulates many essential genes and upregulates numerous inducible TATA-containing genes further indicates that the observed changes occur at the level of transcription activation (47,64). Because one-third of genes deregulated on topo II inactivation are also genes commonly altered by general stress conditions (41), we removed these overlapping genes and conducted functional and structural analyses on genes strictly affected by topo II. This correction allowed uncovering singular traits of genes up- and downregulated by topo II, which could be otherwise masked by those of the genes that commonly respond to general stress. On one side, topo II inactivation downregulates functions mainly related to chromatin remodeling and Pol II transcription, which likely reflects the participation of the enzyme in these processes. On the other side, topo II deactivation upregulates many genes involved in polyamine transport, which are rarely altered in other transcriptome responses. Polyamines are essential for cell growth and regulate chromatin structure by directly affecting DNA topology and nucleosome stability (65). Polyamines also regulate the molecular interactions of topo II (66,67) and stimulate its catalytic activity (68). The activation of polyamine transport may thus reflect a homeostatic response, which attempts to stimulate topo II activity and/or to stabilize chromatin structure. Therefore, contrasting with a functional stress response that imposes a broad self-limiting set of general processes, the functional response to topo II deactivation appears to be specific and driven toward counter-balancing processes.

The singularity of the yeast gene subsets deregulated by topo II becomes more evident when the configuration of their regulatory regions is compared with the genome average. Upregulated genes have a larger promoter size and a wider upstream distribution of TFBS than downregulated ones. No individual transcription factors are associated with the up- or downregulated gene subsets. Thus, rather than by interacting with specific TFs, topo II regulation may operate before TF binding or after TF signaling. The enrichment in divergent transcription and the complementary binding patterns of individual TFs suggest that global aspects of promoter architecture made these genes sensitive to topo II. Other dissimilarities concern the DNA base pair composition of the promoter regions. GC content correlates with double-stranded DNA stability and nucleosome formation potential (61). Compared with the yeast average, downregulated genes present a wider GC depletion upstream the TSS. Violations of PR2 associate to duplex transitions that expose single-stranded DNA (69). Deregulated genes present PR2 profiles that indicate compositional footprints of distinctive DNA unwinding behavior around the TSS.

However, the most revealing differences are in regard to the chromatin structure of deregulated genes. Compared with the yeast gene average, both up- and downregulated groups present a weak nucleosome exclusion region immediately upstream of the TSS. This feature has been observed also in genes with altered transcript levels in *Δtop1 top2-ts* double mutants (25) and may thus reflect an increased sensibility of this configuration to global reduction of topoisomerase activity. Besides this common trait, up- and downregulated genes markedly differ in their nucleosome positioning patterns around their TSS regions. Consistent with their overall lower nucleosome density, upregulated genes have weak −1 and −2 peaks and a strong +1 positioning signal, which may nucleate a barrier-like pattern for downstream nucleosomes (70). Conversely, downregulated genes present well-defined peaks for position −1 and −2 and a weaker signal for nucleosome +1, suggesting that regulation is less dependent on a nucleosomal barrier than on a more subtle coordination of NFR and TF recruitment. Because these positioning patterns are well predicted by SymCurv, the intrinsic DNA bendability is likely to be the main driver for the nucleosome organization. Consequently, sequence constraints of topo II sensitive genes may determine chromatin conformation and its interplay with nuclear components. The fact that nucleosome positioning patterns of upregulated genes differ from those of TATA-containing and general stress upregulated genes corroborate that topo II sensitive genes are structurally distinct from generally inducible genes. Likewise, patterns of downregulated genes are also different from those of TATA-less and general stress downregulated genes.

Analyses of chromatin dynamics show more distinctive traits for genes deregulated by topo II. First, the profiles of appearing and evicted nucleosomes around the TSS are more pronounced than in the genome average. The weak positioning peak for nucleosome +1 in downregulated genes is consistent with the strong appearing nucleosome at this position. Second, an elucidating difference is regarding the association to chromatin remodelers, which is unusually high in downregulated genes and low in the upregulated ones. This strong asymmetry is not observed in TATA-containing genes and stress-regulated genes. The preferential association to chromatin remodelers may reflect the need to actively displace the well-positioned nucleosomes −1 and −2 in the downregulated genes. In turn, remodeler activity may be less relevant in the upregulated group, which has weak nucleosome positional peaks upstream the TSS.

Finally, the histone modification patterns of genes deregulated by topo II inactivation are also distinctive. The downregulated group is enriched in genes that in normal conditions exhibit increased H2AZK14 acetylation at the promoter and H3K4 methylation at the ORF regions. These two modifications are hallmarks of transcriptional activation (71,72), which may thus involve topo II activity. The upregulated group of genes exhibits instead a strong trend to be hypo-methylated and hypo-acetylated, at both their promoter and ORF regions. This modification pattern differs from that of TATA-induced and stress upregulated genes. Histone hypo-acetylation is usually associated to heterochromatic regions (73,74) and correlates with repression of transcription along with H3K36 methylation (75,76). Upregulated genes may thus have a more compacted chromatin structure, which may counteract their fuzzy nucleosome organization. Upregulated genes also present a significant enrichment of the histone variant H2AZ downstream the TSS rather than upstream of it. As H2AZ-containing nucleosomes present high turnover rates (77,78), this trait may relate to the strong +1 positioning signal observed in these genes.

**Figure 7.**
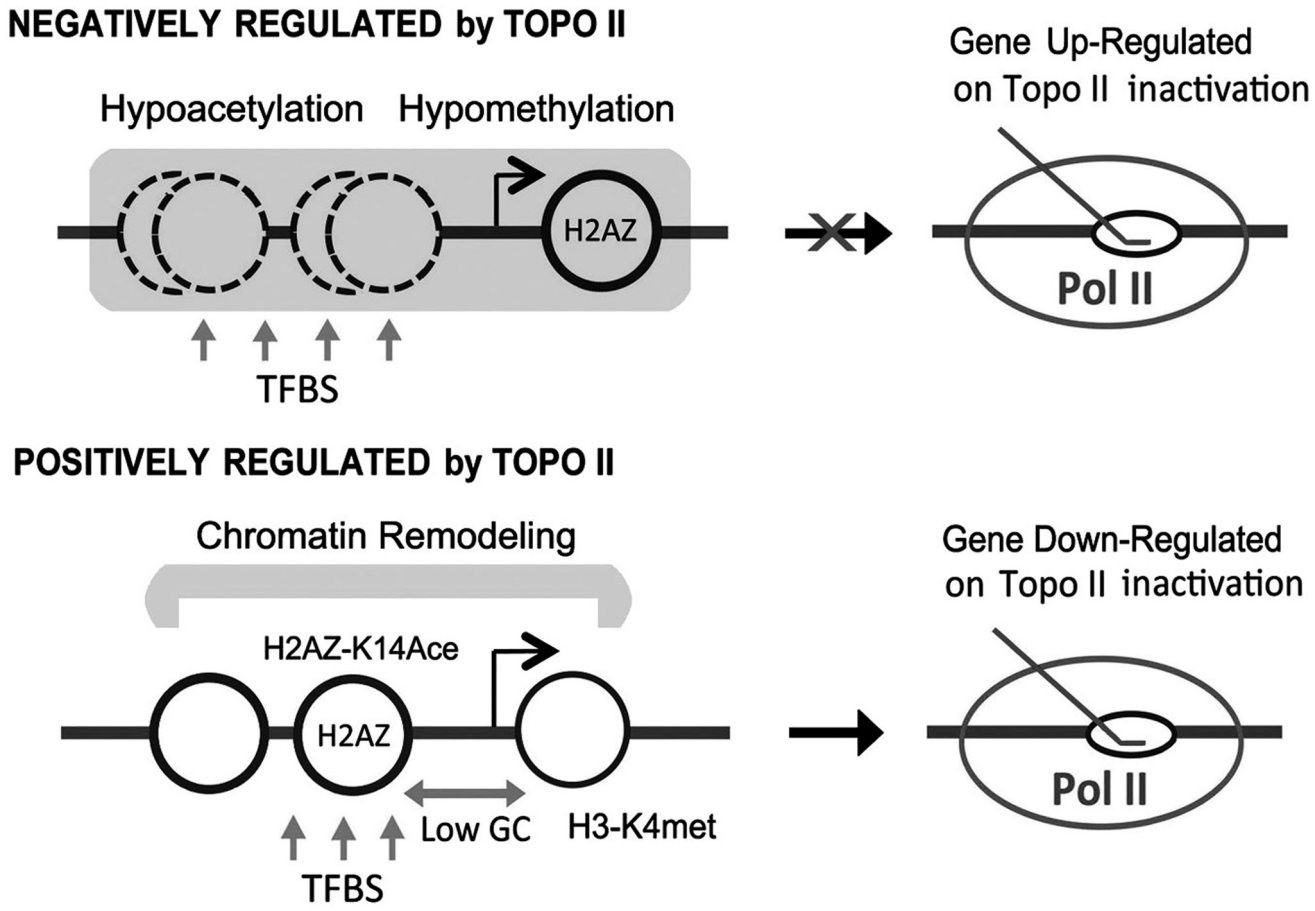
Distinctive promoter architecture of yeast genes regulated by topo II. Most significant features of the regulatory regions of yeast genes positively and negatively regulated by topo II are illustrated.

Figure 7 summarizes the main structural traits and illustrates the singularity of the yeast genes sensitive to topo II deactivation. Remarkably, many of these traits resemble characteristics of genes regulated by topo II in mammalian cells. On one hand, our results show that yeast topo II is a positive regulator of genes with a well-defined promoter architecture and physically associated to chromatin remodeling complexes. A similar context is found in mammalian cells, where topo IIα and topo IIβ isoenzymes interact with chromatin remodelers (30,79), and DNA cleavage activity of topo IIβ occurs at specific gene loci in conjunction with machinery that activates transcription (36,80,81). Yeast genes activated by topo II have a deeper GC depletion at their promoters, and topo IIβ is found to bind preferentially with AT-rich intergenic regions (35). Yeast genes positively regulated by topo II present a significantly increased H3K4 methylation, and topo IIβ is found also to mainly interact with regions containing H3K4 methylation (38). Yeast and mammalian topo II enzymes may thus function in similar environments in conjunction with remodeling complexes to adjust DNA topology for transcriptional activation. On the other hand, our results show that yeast topo II is a negative regulator of highly hypo-acetylated genes with undefined nucleosome positioning, which suggests transcriptional repression by chromatin condensation. Topo II has been long involved in high-order organization of chromatin (9), centromere configuration (82) and genome compaction in sperm cells (83–85). In mammalian cells, topo IIβ is implicated in heterochromatin transitions that depend on histone deacetylase (81). Therefore, yeast topo II and mammalian topo IIβ may use similar mechanisms to repress transcription by inducing or stabilizing condensed chromatin states. Interestingly, many genes repressed by yeast topo II are involved in the transport of polyamines, which favor chromatin condensation. The configuration of these genes may serve thus to both detect and functionally counterbalance a failure of topo II activity.

In summary, our study reveals that yeast topo II is not only the essential decatenase and main relaxase of DNA but that it also plays a regulatory role in the expression of specific genes. Although genes regulated by topo II in yeast are functionally unrelated to those regulated by topo II in mammals, our results suggest that transactions involving chromatin organization and topo II activity may be conserved.

## SUPPLEMENTARY DATA

Supplementary Data are available at NAR Online.

Supplementary Data
