# Supplementary material for: Topoisomerase II regulates yeast genes with singular chromatin architectures
Source: Nucleic Acids Res. 2013 Aug 9;41(20):9243–56. doi: 10.1093/nar/gkt707 (PMC3814376; doi:10.1093/nar/gkt707)
Supplement: Supplementary Data [file supp_41_20_9243__index.html]

Topoisomerase II regulates yeast genes with singular chromatin architectures — Topoisomerase II regulates yeast genes with singular chromatin architectures — Supplementary Data 

# Topoisomerase II regulates yeast genes with singular chromatin architectures

## Supplementary Data

files

**Files in this Data Supplement:**

- Supplementary Data - pdf file
