# Supplementary material for: Topoisomerase II regulates yeast genes with singular chromatin architectures
Source: Nucleic Acids Res. 2013 Aug 9;41(20):9243–56. doi: 10.1093/nar/gkt707 (PMC3814376; doi:10.1093/nar/gkt707)
Supplement: Supplementary Data [file supp_gkt707_nar-01515-f-2013-File009.pdf]

# Supplementary Information

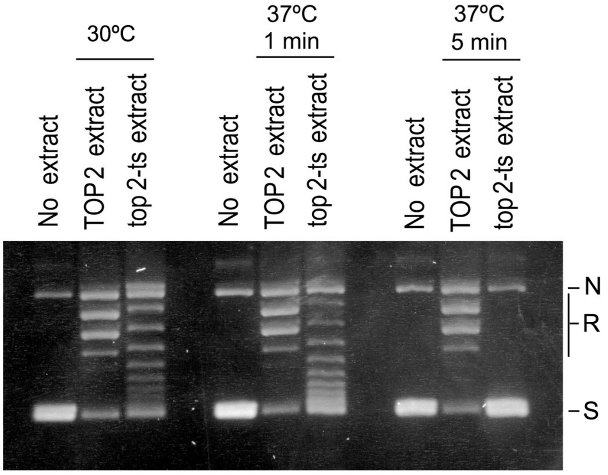


**Figure S1.** Thermal inactivation of topo II activity in *top2-4* yeast mutants. The gel shows DNA relaxation by topo II activity present in extracts from *TOP2* and *top2-ts* yeast cells that lack topoisomerase I. To do this, the strain JCW27 (*TOP2 Δtop1*) and its relative JCW28 (*top2-4 dtop1*) were grown at 30ºC with no thermal shift, or shifted to 37ºC during 1 min and 5 min. Whole cell extracts were obtained by cold glass-bead lysis, cleared by centrifugation and normalized by protein concentration. Diluted extracts were incubated with a supercoiled DNA plasmid in 50 mM Tris–HCl (pH 8), 1 mM EDTA, 150 mM KCl, 8 mM $MgCl_2$, 7 mM 2-mercaptoethanol and 1 mM ATP during 15 min at 30ºC. Reactions were stopped by the addition of SDS to 0.5% and the DNA products examined by agarose gel electrophoresis. S, supercoiled plasmid; N, nicked plasmid; R, relaxed topoisomers.

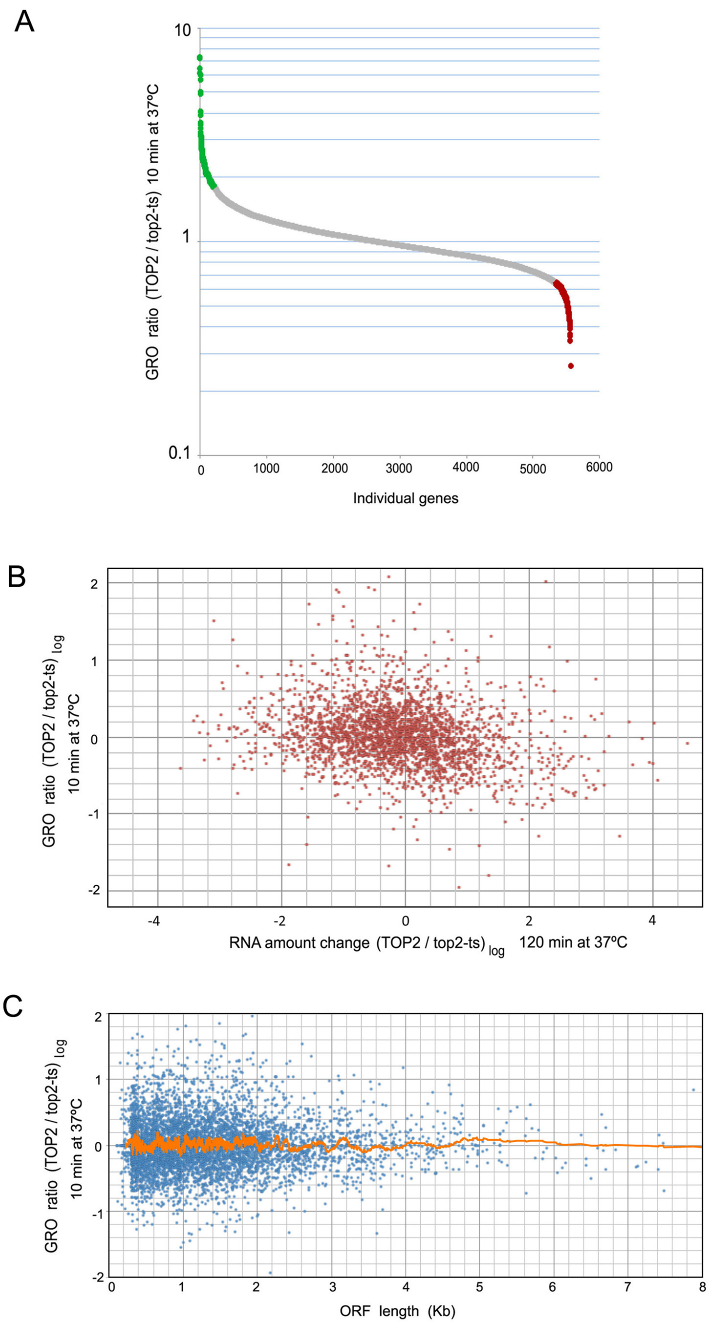


**Figure S2.** Changes of transcription rate of individual yeast genes after 10 min topo II inactivation. **(A)** Scatter plot of transcription alterations upon 10 min inactivation of topo II. Individual yeast genes (x axis) are ordered by the fold changes in transcription frequency (Y axis, log scale), averaged from the three biological replicates of the GRO experiment. 270 genes (~5%) increased their transcription frequency by more than 1.5-fold (green dots); and 158 genes (~3%) decreased it below 0.65-fold (red dots). (**B**) The changes are plotted against the alterations of RNA abundance observed by Joshi et al (2012) after prolonged inactivation of topo II (120 min). (**C**) The changes are plotted against the corresponding ORF length. The orange line is the 100-gene moving average.

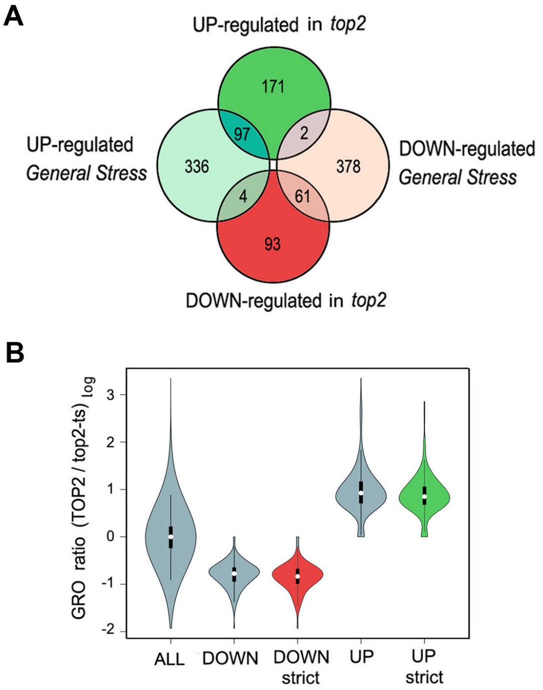


**Figure S3.** Gene subsets deregulated by topo II but not by general stress conditions. **(A)** Overlap analysis of the number of genes deregulated by thermal deactivation of topo II with the environmental stress response (ESR) genes defined by Gasch et al. (2000) **(B)** Comparison of overall GRO values of genes subsets deregulated by topo II deactivation with the strict subsets (173 up- and 97 down-regulated genes) obtained after excluding ESR genes.

## DOWN-Regulated

Chromatin remodeling
mRNA transcription

GO:0006397:mRNA processing
GO:0016568:chromatin modification
GO:0006357:regulation of transcription from RNA polymerase II promoter
GO:0005643:nuclear pore
GO:0006368:transcription elongation from RNA polymerase II promoter
GO:0006338:chromatin remodeling
GO:0030528:transcription regulator activity
GO:0006611:protein export from nucleus
GO:0006409:tRNA export from nucleus
GO:0006609:mRNA-binding (hnRNP) protein import into nucleus
GO:0006607:NLS-bearing substrate import into nucleus
GO:0006610:ribosomal protein import into nucleus
GO:0006608:snRNP protein import into nucleus
GO:0006408:snRNA export from nucleus
GO:0005844:polysome

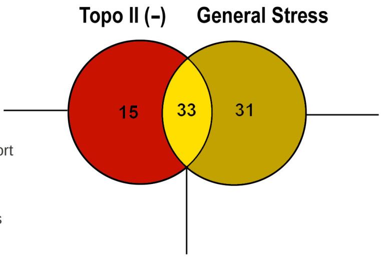


RNA processing

GO:0003723:RNA binding
GO:0006412:translation
GO:0005840:ribosome
GO:0030529:ribonucleoprotein complex
GO:0005730:nucleolus
GO:0003735:structural constituent of ribosome
GO:0006364:rRNA processing
GO:0042254:ribosome biogenesis
GO:0008380:RNA splicing
GO:0022625:cytosolic large ribosomal subunit
GO:0008033:tRNA processing
GO:0030686:90S preribosome
GO:0030687:preribosome large subunit precursor
GO:0042273:ribosomal large subunit biogenesis
GO:0003743:translation initiation factor activity
GO:0006413:translational initiation
GO:0003899:DNA-directed RNA polymerase activity
GO:0005666:DNA-directed RNA polymerase III complex

Cell-cycle
mRNA translation

GO:0003676:nucleic acid binding
GO:0007049:cell cycle
GO:0008652:cellular amino acid biosynthetic process
GO:0004386:helicase activity
GO:0006260:DNA replication
GO:0022627:cytosolic small ribosomal subunit
GO:0006407:rRNA export from nucleus
GO:0019843:rRNA binding
GO:0000027:ribosomal large subunit assembly
GO:0006396:RNA processing
GO:0007064:mitotic sister chromatid cohesion
GO:0042274:ribosomal small subunit biogenesis
GO:0000176:nuclear exosome (RNase complex)
GO:0071038:nuclear polyadenylation-dependent tRNA catabolic process
GO:0006400:tRNA modification
GO:0071051:polyadenylation-dependent snoRNA 3'-end processing
GO:0045047:protein targeting to ER
GO:0071042:nuclear polyadenylation-dependent mRNA catabolic process
GO:0000028:ribosomal small subunit assembly
GO:0005736:DNA-directed RNA polymerase I complex
GO:0000054:ribosomal subunit export from nucleus
GO:0000175:3'-5'-exoribonuclease activity
GO:0000178:exosome (RNase complex)
GO:0006614:SRP-dependent cotranslational protein targeting to membrane

## UP-Regulated

Membrane transport of polyamines

GO:0005773:vacuole
GO:0005215:transporter activity
GO:0005886:plasma membrane
GO:0016021:integral to membrane
GO:0016020:membrane
GO:0046658:anchored to plasma membrane
GO:0015848:spermidine transport
GO:0015847:putrescine transport
GO:0015833:peptide transport
GO:0015606:spermidine transmembrane transport
GO:0015203:polyamine transmembrane transport
GO:0015193:L-proline transmembrane transport
GO:0009898:internal side of plasma membrane
GO:0006885:regulation of pH
GO:0006857:oligopeptide transport
GO:0000501:flocculation via cell wall protein-carbohydrate interaction
GO:0000297:spermine transmembrane transport
GO:0000296:spermine transport
GO:0000148:13-beta-D-glucan synthase complex
GO:0000128:flocculation

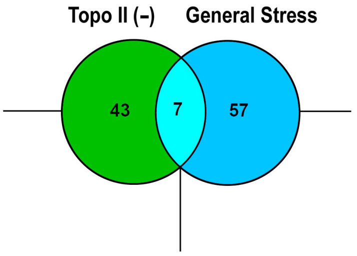


General metabolic regulation

GO:0006094:gluconeogenesis
GO:0009898:internal side of plasma membrane
GO:0006559:L-phenylalanine catabolic process
GO:0006067:ethanol metabolic process
GO:0004737:pyruvate decarboxylase activity
GO:0000949:aromatic amino acid family catabolic process to alcohol via Ehrlich pathway
GO:0000328:fungal-type vacuole lumen

General metabolic regulation
Protein degradation

GO:0030479:actin cortical patch
GO:0005739:mitochondrion
GO:0070682:proteasome regulatory particle assembly
GO:0070469:respiratory chain
GO:0045721:negative regulation of gluconeogenesis
GO:0045333:cellular respiration
GO:0034515:proteasome storage granule
GO:0034045:pre-autophagosomal structure membrane
GO:0032947:protein complex scaffold
GO:0032197:transposition RNA-mediated
GO:0031930:mitochondria-nucleus signaling pathway
GO:0030665:clathrin coated vesicle membrane
GO:0030276:clathrin binding
GO:0019774:proteasome core complex beta-subunit complex
GO:0019773:proteasome core complex alpha-subunit complex
GO:0010499:proteasomal ubiquitin-independent protein catabolic process
GO:0008645:hexose transport
GO:0008541:proteasome regulatory particle lid subcomplex
GO:0008540:proteasome regulatory particle base subcomplex
GO:0006635:fatty acid beta-oxidation
GO:0006569:tryptophan catabolic process
GO:0006513:protein monoubiquitination
GO:0006458:'de novo' protein folding
GO:0006123:mitochondrial electron transport cytochrome c to oxygen
GO:0006122:mitochondrial electron transport ubiquinol to cytochrome c
GO:0006109:regulation of carbohydrate metabolic process
GO:0005905:coated pit
GO:0000407:pre-autophagosomal structure
GO:0000147:actin cortical patch assembly

**Figure S4.** Gene ontology categories of functions up- and down regulated by topo II deactivation and by general stress. Diagrams indicate categories exclusively altered for each of the two conditions and in common to both. The ontology categories are obtained by applying a p-value threshold of $p<0.001$.

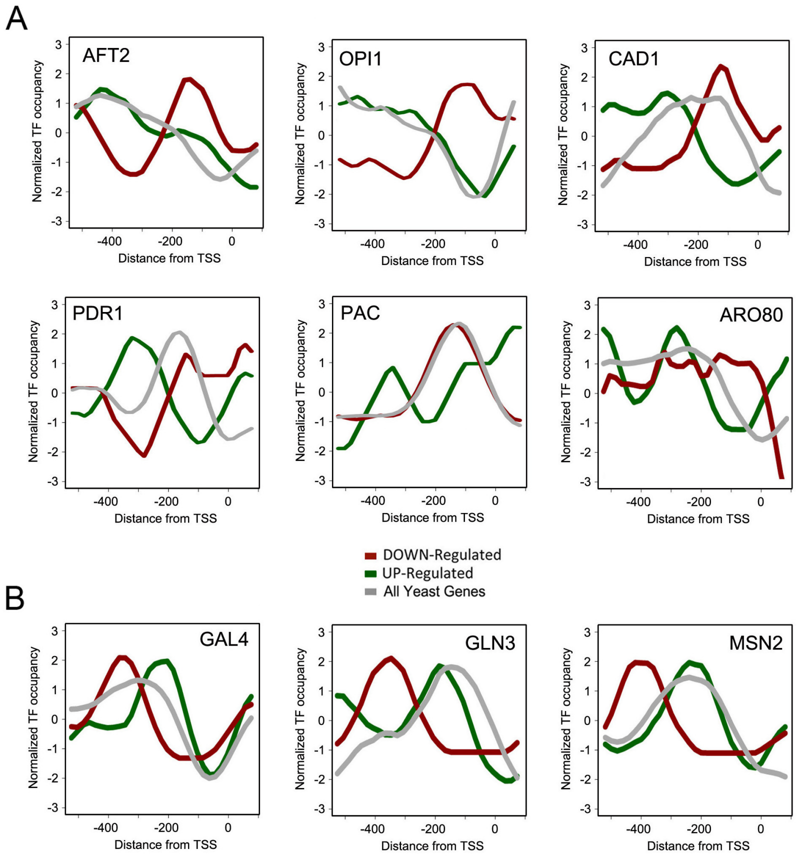


**Figure S5.** Binding site distribution of individual transcription factors**. (A)** Transcription factors whose pattern of binding sites enrichment was proximal to the TSS in down-regulated genes, while it was farther upstream in up-regulated genes. **(B)** Transcription factors whose pattern of binding sites enrichment was proximal to the TSS in up-regulated genes and farther upstream in down-regulated ones. Occurrence of each binding site in a region of -500 nts flanking the TSS of each gene was calculated in bins of 20 bps for each case. Normalization was performed as z-score, meaning that from each value the mean was subtracted and the remainder was divided over the standard deviation.

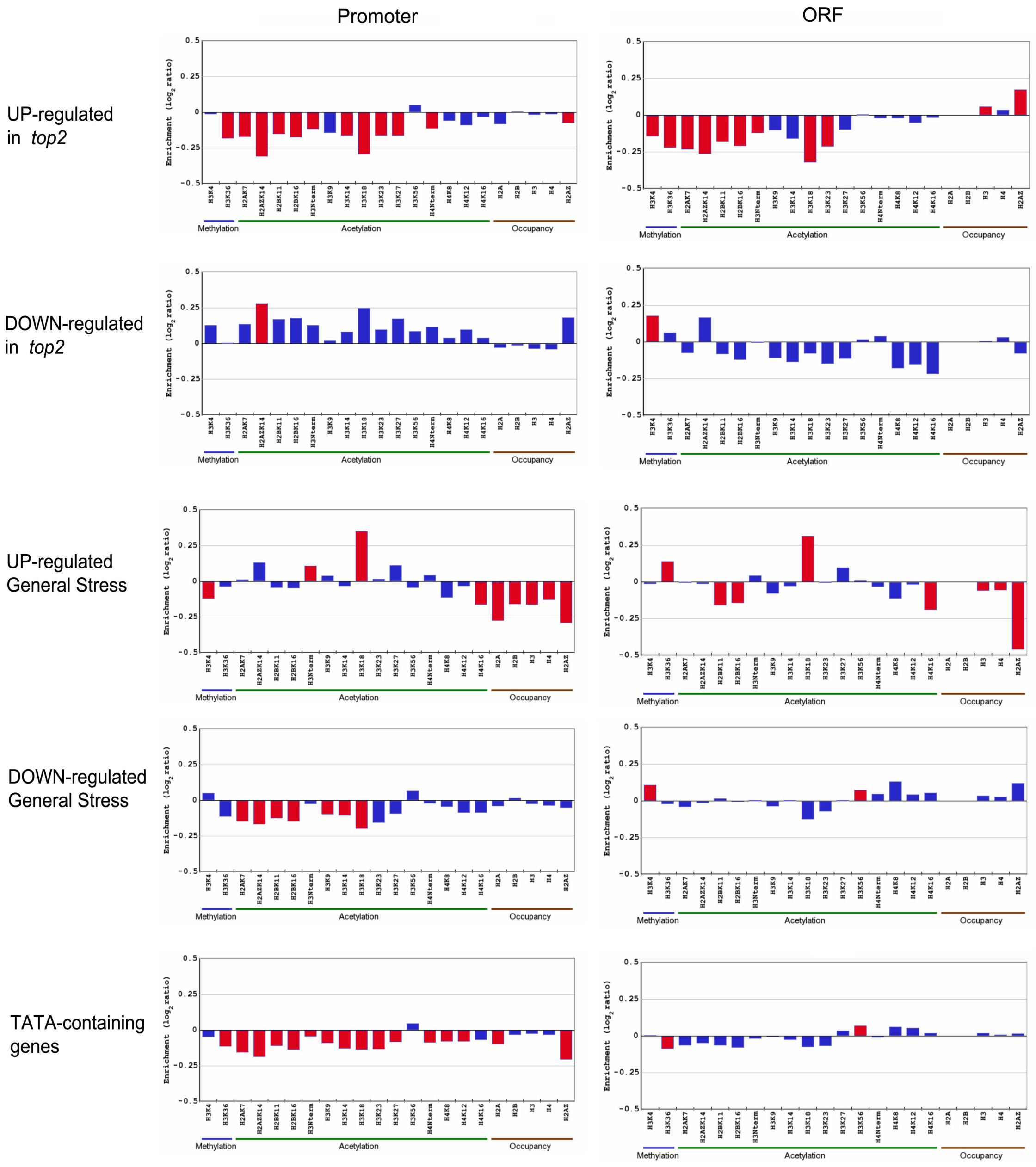


**Figure S6.** Comparison of histone modification patterns of genes deregulated by topo II inactivation, by general stress conditions and TATA containing genes. Histone modification enrichments were analyzed in ChromatinDB. In each case, relative enrichment (gene subset average compared to the total yeast average) was evaluated based on a Wilcoxon rank sum test for both gene promoters and the ORF regions. Red stars denote significant enrichment or depletion (P-value <0.001).

**Table S1. Yeast genes Up- and Down-regulated upon 10 min inactivation of topo II**

Genes commonly altered in general stress conditions are excluded
Fold change is the ratio of transcription rate of *top2-ts/TOP2* strains observed by GRO

## UP-Regulated
(173 genes)

| NAME | FOLD |
|---|---|
| YOR348C | 7.13 |
| YCL025C | 7.10 |
| YPR015C | 7.03 |
| YGL262W | 6.03 |
| YGR138C | 5.60 |
| YJL037W | 5.35 |
| YGR035C | 4.79 |
| YMR017W | 4.66 |
| YPR156C | 4.49 |
| YAL005C | 4.21 |
| YEL071W | 4.06 |
| YLR307W | 3.87 |
| YLR414C | 3.84 |
| YDR034W-B | 3.52 |
| YCR061W | 3.44 |
| YBR198C | 3.39 |
| YLR121C | 3.24 |
| YLR445W | 3.13 |
| YPR157W | 3.08 |
| YPL003W | 2.98 |
| YIL066C | 2.91 |
| YNL231C | 2.84 |
| YGR053C | 2.82 |
| YDR216W | 2.73 |
| YKL161C | 2.69 |
| YFL053W | 2.67 |
| YJR149W | 2.64 |
| YEL070W | 2.63 |
| YGR236C | 2.59 |
| YHR092C | 2.59 |
| YJR078W | 2.56 |
| YLR099C | 2.49 |
| YGR055W | 2.48 |
| YOR375C | 2.48 |
| YPR037C | 2.48 |
| YFL061W | 2.47 |
| YMR011W | 2.47 |
| YDR009W | 2.44 |
| YGL045W | 2.44 |
| YLR132C | 2.43 |
| YOR208W | 2.39 |
| YPL014W | 2.33 |
| YIR017C | 2.32 |
| YLL062C | 2.29 |
| YPR007C | 2.29 |
| YOL113W | 2.28 |
| YGR153W | 2.27 |
| YLR377C | 2.27 |
| YGR131W | 2.26 |
| YNL332W | 2.26 |
| YLR437C | 2.25 |
| YOR391C | 2.21 |
| YJL052W | 2.20 |
| YOL089C | 2.20 |
| YKL086W | 2.16 |
| YOR387C | 2.16 |
| YBR298C | 2.15 |
| YGL096W | 2.15 |
| YNL128W | 2.15 |
| YGL184C | 2.14 |
| YLR119W | 2.14 |
| YPL222W | 2.11 |
| YLR376C | 2.09 |
| YDL169C | 2.07 |
| YLL061W | 2.07 |
| YLR303W | 2.06 |
| YLR283W | 2.05 |
| YAL053W | 2.04 |
| YFL058W | 2.03 |
| YIR041W | 2.03 |
| YPL027W | 2.03 |
| YGR189C | 2.01 |
| YLR211C | 2.01 |
| YOL016C | 2.00 |
| YBR177C | 1.99 |
| YPR078C | 1.99 |
| YDR259C | 1.98 |
| YHR175W | 1.98 |
| YNR056C | 1.98 |
| YPR006C | 1.98 |
| YER142C | 1.95 |
| YBR295W | 1.94 |
| YGL006W | 1.94 |
| YGL209W | 1.94 |
| YKR093W | 1.93 |
| YLR137W | 1.93 |
| YNL335W | 1.92 |
| YPL088W | 1.91 |
| YJL082W | 1.89 |
| YPL111W | 1.88 |
| YLR023C | 1.86 |
| YLR125W | 1.86 |
| YLR237W | 1.86 |
| YPR193C | 1.86 |
| YJR154W | 1.85 |
| YMR252C | 1.85 |
| YER098W | 1.84 |
| YLR174W | 1.84 |
| YDR043C | 1.83 |
| YLR345W | 1.81 |
| YNL014W | 1.81 |
| YDL125C | 1.80 |
| YDL010W | 1.79 |
| YOR389W | 1.79 |
| YLR313C | 1.78 |
| YER145C | 1.77 |
| YLR164W | 1.77 |
| YLR380W | 1.77 |
| YJL144W | 1.76 |
| YLR203C | 1.76 |
| YBR284W | 1.75 |
| YER044C | 1.75 |
| YLR390W | 1.75 |
| YNR057C | 1.75 |
| YIR032C | 1.74 |
| YJL170C | 1.74 |
| YGR180C | 1.73 |
| YJL026W | 1.73 |
| YMR084W | 1.73 |
| YBR203W | 1.72 |
| YDR078C | 1.72 |
| YGL125W | 1.72 |
| YHR002W | 1.72 |
| YPR165W | 1.72 |
| YLR460C | 1.71 |
| YJL172W | 1.69 |
| YMR322C | 1.69 |
| YER026C | 1.68 |
| YLR241W | 1.68 |
| YCL038C | 1.67 |
| YDL247W | 1.67 |
| YHL016C | 1.67 |
| YLR326W | 1.67 |
| YNL333W | 1.67 |
| YPL221W | 1.67 |
| YGL048C | 1.66 |
| YNL175C | 1.66 |
| YFL059W | 1.65 |
| YJL132W | 1.65 |

| | |
|---|---|
| YLR187W | 1.65 |
| YLR250W | 1.65 |
| YLR329W | 1.65 |
| YPR198W | 1.65 |
| YAL054C | 1.64 |
| YER015W | 1.64 |
| YKR075C | 1.64 |
| YLR176C | 1.64 |
| YDR497C | 1.63 |
| YLR390W-A | 1.63 |
| YNR045W | 1.63 |
| YGR097W | 1.62 |
| YMR272C | 1.62 |
| YPR194C | 1.62 |
| YHL047C | 1.61 |
| YER060W-A | 1.60 |
| YLR454W | 1.60 |
| YMR306W | 1.60 |
| YLR315W | 1.59 |
| YMR269W | 1.59 |
| YAL001C | 1.58 |
| YJL223C | 1.58 |
| YLR193C | 1.58 |
| YAL030W | 1.57 |
| YLR271W | 1.57 |
| YNL294C | 1.57 |
| YEL065W | 1.55 |
| YLR310C | 1.55 |
| YPR001W | 1.55 |
| YBR208C | 1.54 |
| YDL238C | 1.54 |
| YBR213W | 1.53 |
| YMR010W | 1.52 |

**DOWN-Regulated**
(97 genes)

| | |
|---|---|
| YBR141C | 0.65 |
| YDL179W | 0.65 |
| YGR251W | 0.65 |
| YBR154C | 0.64 |
| YBR262C | 0.64 |
| YIR033W | 0.64 |
| YER123W | 0.63 |
| YGL086W | 0.63 |
| YGR083C | 0.63 |
| YIL104C | 0.63 |
| YKR044W | 0.63 |
| YML023C | 0.63 |
| YCL029C | 0.62 |
| YGR195W | 0.62 |
| YHR196W | 0.62 |
| YJR069C | 0.62 |
| YKR026C | 0.62 |
| YOL141W | 0.62 |
| YPL239W | 0.62 |
| YDL049C | 0.61 |
| YDR280W | 0.61 |
| YDR441C | 0.61 |
| YHR084W | 0.61 |
| YHR169W | 0.61 |
| YDL033C | 0.60 |
| YGR081C | 0.60 |
| YGR119C | 0.60 |
| YGR207C | 0.60 |
| YGR267C | 0.60 |
| YKL040C | 0.60 |
| YMR108W | 0.60 |
| YNL145W | 0.60 |
| YOR287C | 0.60 |
| YBL016W | 0.59 |
| YBR119W | 0.59 |
| YBR244W | 0.59 |
| YBR265W | 0.59 |
| YGR211W | 0.59 |
| YOR188W | 0.59 |
| YDL208W | 0.58 |
| YHR005C | 0.58 |
| YPL012W | 0.58 |
| YAR015W | 0.57 |
| YDL043C | 0.56 |
| YLL018C-A | 0.56 |
| YLR056W | 0.56 |
| YOR006C | 0.56 |
| YER115C | 0.55 |
| YGR136W | 0.55 |
| YIL091C | 0.55 |
| YJR115W | 0.55 |
| YKL041W | 0.55 |
| YNL299W | 0.55 |
| YPR065W | 0.55 |
| YGR239C | 0.54 |
| YJR112W | 0.54 |
| YMR014W | 0.54 |
| YOL093W | 0.54 |
| YPR119W | 0.54 |
| YCR072C | 0.53 |
| YJL136C | 0.53 |
| YLR027C | 0.53 |
| YBR121C | 0.52 |
| YFL026W | 0.52 |
| YHR053C | 0.52 |
| YJL157C | 0.52 |
| YJL193W | 0.52 |
| YML030W | 0.52 |
| YLR359W | 0.51 |
| YAR002W | 0.50 |
| YGR208W | 0.50 |
| YBR083W | 0.49 |
| YCL026C-B | 0.49 |
| YDR281C | 0.49 |
| YGL032C | 0.48 |
| YJL056C | 0.48 |
| YBR296C | 0.47 |
| YKR071C | 0.47 |
| YMR134W | 0.47 |
| YCL055W | 0.44 |
| YGR054W | 0.44 |
| YHR086W | 0.44 |
| YIL015W | 0.44 |
| YEL003W | 0.41 |
| YKL120W | 0.41 |
| YMR181C | 0.40 |
| YBR028C | 0.38 |
| YOR136W | 0.38 |
| YBL002W | 0.36 |
| YHL017W | 0.35 |
| YOR226C | 0.35 |
| YCL036W | 0.34 |
| YFR032C-A | 0.34 |
| YNL037C | 0.32 |
| YNR044W | 0.31 |
| YCL018W | 0.29 |
| YCL027W | 0.26 |
